# Integrating Prevention and Response at the Crossroads of Henipavirus Preparedness, Hendra@30 Conference, 2024

**DOI:** 10.3201/eid3201.250979

**Published:** 2026-01

**Authors:** Kim Halpin, Raúl Gómez Román, Emmie de Wit, Alison J. Peel, Jonathan H. Epstein, Jennifer Barr, Sarah J. Edwards, Melanie N. Tripp, Berta Blanch-Lázaro, Belinda Linnegar, Gervais Habarugira, Glenn A. Marsh, Danielle Anderson, Li-Yen Chang, Wanda Markotter

**Affiliations:** CSIRO Australian Centre for Disease Preparedness, Geelong, Victoria, Australia (K. Halpin, J. Barr, S.J. Edwards, M.N. Tripp, B. Blanch-Lázaro, G. Habarugira, G.A. Marsh); Independent Science Consultant, Ensenada, Mexico (R. Gómez Román); University of Sydney, Camperdown, New South Wales, Australia (A.J. Peel); One Health Science, New York, New York, USA (J.H. Epstein); Monash University, Melbourne, Victoria, Australia (M.N. Tripp); Griffith University, Nathan, Queensland, Australia (B. Linnegar); Victorian Infectious Disease Reference Laboratory, Melbourne (D. Anderson); Universiti Malaya, Kuala Lumpur, Malaysia (L.-Y. Chang); University of Pretoria, Pretoria, South Africa (W. Markotter)

**Keywords:** Hendra virus, Nipah virus, henipavirus, viruses, Hendra@30, Australia

## Abstract

Diseases caused by henipaviruses, exemplified by Hendra virus and Nipah virus, pose a serious risk to public health because of their epidemic potential and high case-fatality rates and the paucity of medical countermeasures to mitigate them. In December 2024, a group of 150 scientists from 16 countries convened in Geelong, Victoria, Australia, to mark the 30th anniversary of the discovery of Hendra virus. The Hendra@30 conference built upon its predecessor conference held in 2019 in Singapore, Nipah@20, by expanding its program across broader disciplines and integrating sessions on human sociology and disease ecology into the main scientific discussions. We describe key highlights from Hendra@30 and reflect on 4 key elements that have advanced henipavirus research and medical countermeasures research and development. We propose that integrating bat ecology into henipavirus research blueprints will enable development of ecologic countermeasures that prevent spillover and will complement existing preparedness and response efforts with evidence-based prevention strategies.

Henipaviruses, exemplified by Hendra virus (HeV) and Nipah virus (NiV), represent critical threats to global health security, given their broad host geographic range, repeated spillover into humans and domestic animals, high case-fatality rates (CFR; 57% CFR for HeV (*1*) and 80% CFR for NiV [*2*]), and limited medical countermeasures, creating substantial challenges for outbreak preparedness. Since its emergence in 1994, HeV has caused devastating outbreaks in Australia with infection primarily in horses and humans; there were >105 equine cases in which the animals died or were euthanized and 7 human cases, including 4 deaths (*3*). NiV was identified in 1998 after it caused a large outbreak in Malaysia and Singapore that affected pigs and humans, resulting in 110 human deaths (*1*). Since then, NiV outbreaks have been reported in Bangladesh and India, with near-annual human outbreaks in Bangladesh since 2001. An outbreak consistent with NiV infection also occurred in the Philippines, although full viral sequence and isolates were not obtained (*1*).

The scientific community has recently mobilized efforts to address those challenges through policy frameworks, research roadmaps, investments in vaccines and therapeutics, and collaborative scientific exchange. The World Health Organization (WHO) prioritized *Henipavirus nipahense* as a prototype pathogen within the *Paramyxoviridae* family (*4*). In parallel, 15 subject matter experts published an update to the 2019 WHO research and development (R&D) roadmap for accelerating the development of medical countermeasures to enable effective and timely responses to NiV outbreaks (*5*). The WHO South-East Asia Regional Office (SEARO) synthesized those global research and policy initiatives into a strategy for the prevention and control of NiV in the Southeast Asia region, developed through expert consultation and published in 2023 (*6*). CSIRO and the Coalition for Epidemic Preparedness Innovations hosted the Hendra@30 Henipavirus International Conference in Geelong, Victoria, Australia, for 150 scientists from 16 countries (*7*); the conference marked the 30th anniversary of the first recognized outbreak of HeV disease (*1*,*3*).

The Hendra@30 conference provided a forum to reflect on the history of henipavirus spillovers, to review 3 decades of scientific findings, and to understand the current challenges in developing diagnostics, therapeutics, and vaccines against henipaviruses in general (Table). Whereas its predecessor conference, Nipah@20, held in 2019 in Singapore, focused mostly on advances in virology and medical countermeasures (*8*), Hendra@30 expanded its program to incorporate bat ecology and behavioral determinants of virus transmission, as well as landscape and climatic drivers of spillover. That interdisciplinary approach brought disease ecologists and social scientists into scientific discussions alongside virologists, veterinarians, and public health experts. In addition to the scientific presentations, Hendra@30 included a tour of the local laboratory, a field visit to the Geelong flying fox colony, and a dedicated session featuring survivors of HeV and NiV outbreaks who shared their personal experiences with the viral diseases and how the outbreaks impacted their families and livelihoods. One survivor described her ongoing struggles with neurologic sequelae, 16 years after HeV infection. The daughter of a patient who succumbed to NiV illness has pursued a career in science, focusing her research on the virus that profoundly affected her family. Last, the first veterinarian to encounter cases of HeV during the initial outbreak shared insights into the emotional challenges faced while addressing the loss of both horses and human lives. Those shared experiences added important dimensions, ensuring that the ecologic context and the human impact of henipaviruses remained central to the meeting’s scientific discussions.

**Table:** Summary of Hendra@30 conference program sessions, 2024*

Day	Session	No. presentations
1	Visit to Geelong flying-fox colony	
2	Welcome	4
	History of Hendra virus	4
	Personal stories	2
	Disease ecology	8
	Rapid oral session talks (accompanying poster presentations)	16
3	Surveillance	6
	Behavioral determinants of henipavirus transmission	3
	Diagnostics	4
	Virology and immunology	8
	Bat infection studies	3
4	Pathogenesis	7
	Vaccines	7
	Therapeutics	5
	Panel discussion: WHO Nipah Virus Roadmap and the new WHO Paramyxovirus CORC–5 panelists and 2 moderators	
5	Tour of CSIRO Australian Centre for Disease Preparedness	

*CORC, Collaborative Open Research Consortium; WHO, World Health Organization.

All countries that have had henipavirus or henipa-like outbreaks (Australia, Bangladesh, India, Malaysia, the Philippines, and Singapore) were represented at the conference; their delegations accounted for 66% (99/150) of all attendees. Reflecting the conference’s commitment to inclusive leadership, the organizing committee comprised three quarters women (17/23 [74%]) and more than one third early-career scientists (8/23 [35%]) and prioritized diverse representation among speakers. To encourage broad participation by the henipavirus research community, organizers provided travel grants to 14 emerging scholars from low- and middle-income countries.

The proceedings from the conference are now publicly available (*7*). Here, we highlight 4 key elements from work presented at the conference and from published work that have substantially advanced henipavirus R&D since 2020 and will likely have a profound impact on henipavirus prevention, preparedness, and response (PPR) efforts over the next 5 years.

## Key Takeaways

### Progress in the Development of Henipavirus Medical Countermeasures

In the 5 years between the Nipah@20 and Hendra@30 conferences, SARS-CoV-2 emerged, and the COVID-19 pandemic was declared. The urgent need for COVID-19 medical countermeasures resulted in the advancement of several diagnostics, vaccines, and therapeutics that will likely advance henipavirus R&D (*9*). For instance, now that several COVID-19 mRNA vaccines are approved for either emergency or routine use in various countries, regulatory familiarity with mRNA platforms could simplify review of mRNA henipavirus vaccines. Preclinical and phase I clinical data for mRNA-1215 were presented, describing early preclinical evidence on cross-protection against NiV and HeV infections. Cross-protection against heterologous henipaviruses had been previously observed with other vaccine platforms, including the HeV protein-subunit and adjuvanted vaccine licensed for use in horses in Australia (*10*). Presenters reviewed the history of that vaccine along with the continued clinical development for human use, including the prospect of delivery via microneedle patches. Although clinical data in humans were presented only for the mRNA vaccine platform, additional veterinary and preclinical data from other vaccine approaches were also reported, including data in foals from mares immunized with the Equivac HeV protein subunit vaccine; data from mice immunized with a polyphosphazene adjuvanted microneedle patch vaccine based on the HeV protein subunit platform; data in Syrian golden hamsters immunized with a Nipah viral replicon vaccine lacking the NiV fusion (F) protein; data in mice and ferrets immunized with self-amplifying, replicon RNA Nipah vaccines, delivered in lipid inorganic nanoparticles; and data in African green monkeys immunized with single-cycle, recombinant vesicular stomatitis virus-based vaccine expressing the NiV glycoprotein (G).

Several small molecules and therapeutics against henipaviruses are under development, mostly in preclinical development with limited clinical data (*9*). Data were presented at the Conference for novel nanobodies (DS90), new combinations of small molecules (dexamethasone and remdesivir), new methods for the discovery and screening of compounds with in vitro antiviral activity, and immunoglobulins recovered from antibody-secreting cells isolated from humans vaccinated with mRNA-1215. A systematic review has identified well designed clinical efficacy trials and in vivo pharmacokinetic and pharmacology studies as 2 bottlenecks needed to move products down the clinical pipeline (*11*).

Overall, Hendra@30 hosted 16 scientists presenting data on medical countermeasures. However, much of the progress in the development of such countermeasures has also been the result of progress in other areas of henipavirus R&D, including understanding the viruses at the molecular level.

### Progress in Understanding the Molecular Mechanisms of the Viruses

The conference offered 18 presentations on studies of viral infection mechanisms across virology/immunology, pathogenesis, and rapid oral sessions, including several studying the nuclear trafficking of henipavirus proteins. The mechanisms to enter the nucleus and associated functions such as viral budding (in matrix protein) and immune modulation (in W protein) were discussed in several presentations. Those functions are often important for viral pathogenesis, and therefore, unravelling those pathways could provide novel drug targets (*12*).

The G and F proteins expressed on the surface of infected cells are a target for the immune system and, hence, medical countermeasures. Cross-reactivity between HeV and NiV has been identified for certain mAbs (including m102.4 and 5B3) against those proteins. Given the recent expansion of henipaviruses and parahenipaviruses, experts discussed the diversity of the G and F proteins across those genera, which holds implications for potential broad-spectrum vaccines and mAbs targeting those proteins.

A second HeV genotype was identified in 2021 (*13*,*14*); at the conference, 2 presentations focused on how that genotype might compare to HeV-g1. One study revealed key differences in disease outcomes from experimental challenge in African green monkeys; HeV-g2–infected animals showed reduced severity of respiratory and neurologic disease compared with the original genotype, leading to improved survival outcomes. The molecular mechanism behind those differences is unclear but may be linked to decreased replication efficiency of HeV-g2, which was described in both presentations, and reduced ability to inhibit the interferon induction response, described in the second presentation.

Studying henipaviruses in a standard animal model, African green monkeys, enabled researchers to examine pathogenesis, including the ways the virus reaches the central nervous system; histopathology suggests the blood–brain barrier or the olfactory bulb. Other in vitro and in vivo models in use explore neuropathogenesis using cerebral organoids and a hamster model of infection. Multiple conference presentations covered various aspects of viral infection mechanisms. That field still has gaps in research, including the extent to which the mechanisms of infection vary across henipavirus genotypes and species. Understanding mechanisms of infection is important, given the known diversity of the viruses in the henipavirus genus.

### Progress in Understanding Henipavirus Genetic Diversity

Understanding of henipavirus diversity has expanded dramatically through metagenomic and metatranscriptomic sequencing. Although published studies have been dominated by detections of henipa-like viruses in shrews and rodents (genus *Parahenipavirus*) (*15**,**16*), dozens of new bat henipaviruses were presented at the conference. First, data were presented on the largest survey of bat henipaviruses from Australian flying foxes, incorporating samples collected during 2018–2021 from sites in southeast Queensland and northeast New South Wales, Australia. That work identified 24 new henipavirus species, all with complete or near-complete genomes, and revealed 3 distinct henipavirus clades. Clade 1 henipaviruses included 4 of the 5 existing known henipaviruses (Hendra, Nipah, Cedar, and Ghana viruses), whereas clade 2 included the remaining known henipaviruses (Angavokely virus, discovered in urine collected from fruit bats in Madagascar [*17*]), and Salt Gully virus, the discovery of which was presented at the conference), plus 5 new species with complete genomes. Clade 3 comprised an entirely novel clade of henipaviruses with 11 complete genomes, 5 near-complete genomes, and 3 partial L genes. Only a few of those newly identified viruses have been isolated as of 2025.

Additional evidence of remarkable diversity emerged from studies on *Rousettus* spp. Egyptian rousette bats in South Africa; 18 putative henipavirus species were identified in those studies. Efforts to understand how genetic diversity translates into antigenic diversity include the creation of a library of soluble G and F proteins from various henipaviruses, including the Langya virus and Angavokely virus F proteins, yielding data on the multimeric diversity of these proteins. Overall, despite major advances in characterizing the extensive genetic diversity of henipavirus and related viruses, particularly those in Australia and South Africa, the implications for cross-species transmission and potential for disease emergence of these new species remain poorly understood.

### Progress in Understanding Henipaviruses in the Context of Bat Ecology and Bat Health

Hendra@30 featured 20 oral and poster presentations examining the ecologic pathways of henipavirus transmission from bats to spillover hosts (*7*). Sessions covered disease ecology (viral load and diversity, community ecology, bridging animal hosts, and anthropogenic drivers of spillover), surveillance (in bats, humans, and horses), behavioral determinants of transmission (from bats to animals and humans), and within-host infection dynamics via experimental infection studies in *Pteropus*, *Rousettus*, and *Artibeus* spp. bats.

A unifying theme of the presentations was the connection between habitat preservation, bat health, and spillover risk, building upon published work demonstrating that HeV spillover is driven by rapid environmental changes (*18*). Research presented used climatic, ecologic, land cover, and flowering data to generate Bayesian network models to accurately predict clusters of HeV spillovers over a 25-year period in Australia. Those studies demonstrated that flying foxes are responding to habitat loss by persistently adopting behaviors previously observed only during climate-driven periods of acute nutritional stress. As a result, flying foxes are losing their nomadism and increasingly remaining in urban and agricultural areas where horse populations have a higher density, increasing spillover risk. Yet when remnant habitat flowered abundantly, providing pulses of natural nectar, flying foxes returned to their natural nomadic behaviors. Every time the increasingly rare flowering occurred during the study period, risk was mitigated and no spillovers occurred. Although spillover can be prevented by vaccinating horses with HeV vaccine, its adoption is declining. Therefore, the behavioral responses of flying foxes to flowering suggest that strategic habitat restoration of species that provide food during periods of resource limitation could sustainably mitigate spillover risk by supporting bat health and reducing contact with spillover hosts.

Conference presentations also built on complementary studies that have linked food shortages with viral shedding whereby viral excretion occurs at higher prevalences, at higher viral loads, and across a broader viral diversity following acute food shortages (*19*,*20*). Those observations suggest that bats live on an energetic edge, and those periods of shortage result in bats experiencing allostatic overload, i.e., when insufficient available energy (i.e., food) forces diversion of energy away from immunity, which reduces suppression of viral infections and increases viral shedding (*21*).

In Bangladesh, Indian flying foxes (*Pteropus medius*) have been found to prefer roosting in forest fragments near higher human population density in the country, rather than intact forest (*22**,**23*). Removal of those timber trees can disrupt bat colonies and lead to colony dispersal. According to a study presented at the conference, 75% of roosts were affected by tree cutting; bat population was an average of 1,700 bats per roost. Bat populations declined at 4 locations, by 63%, 70%, 54% and 20%, from 2021 to 2024. Raw date palm sap, the primary route of zoonotic transmission of NiV in Bangladesh, is not the bat’s natural food source but is available during winter months when other food is scarce. NiV outbreaks were most likely to occur in winter months, coinciding with date palm sap cultivation (*24*).

Collectively, ecologic insights suggest that similar mechanisms might drive henipavirus spillover risk across multiple systems. Scientists in Bangladesh and India have emphasized the need to protect bat habitats and maintain consistent access to natural dietary sources to reduce NiV spillovers (*25*). Ecologic countermeasures that maintain and restore natural bat foraging habitats could also provide, pending local ecologic and socioeconomic context validation, a generalizable, sustainable approach to reducing henipavirus spillover risk in other areas at risk by supporting bat health and minimizing contact with domestic animals and humans.

## Closing Discussions

Both Nipah@20 and Hendra@30 included closing panel discussions that contextualized henipavirus R&D within a global public health perspective. The panel at Hendra@30 specifically addressed the 2024 update of the Nipah R&D roadmap (*5*). The original goal of the roadmap was to develop effective medical countermeasures, including diagnostics, therapeutics, and vaccines, by 2030. The panelists highlighted the enthusiasm and ambitious timelines of the expert group involved. They emphasized the importance of securing funding for R&D, addressing cross-cutting issues, overcoming access and implementation hurdles, and navigating regulatory challenges during outbreaks. Shortly after updating the roadmap, WHO shifted its R&D strategy; the shift in strategy resulted from extensive consultations during 2022–2024 and was influenced by the Coalition for Epidemic Preparedness and Innovations–driven 100-day mission (*26*), which emphasized rapid response to new epidemic threats through the proactive development of prototype vaccines for priority pathogens. Consequently, the WHO strategy prioritizes preparedness and response alongside prevention efforts (*4*). In contrast to earlier pathogen prioritization efforts, which listed 10 priority diseases, including henipaviruses, the new framework identified >30 priority pathogens, of which NiV remains notable within the *Paramyxoviridae* family as both a priority and a prototype pathogen. The designation highlighted NiV as both a representative threat and a basis for potential vaccine development.

During the Hendra@30 panel discussion, WHO’s new approach raised questions, which were discussed. Panelists also introduced the Paramyxovirus Collaborative Open Research Consortium (CORC), cohosted by WHO and the Indian Council of Medical Research, and emphasized its openness to global researchers and its role in addressing knowledge gaps, ensuring equitable access to medical countermeasures, and strengthening community trust. However, several concerns arose; those included the complexity, speed, and governance of the CORC’s operationalization, funding sources, and R&D decision mechanisms. Panelists agreed that aligning Nipah roadmap priorities with the CORC paramyxovirus initiative could benefit overall henipavirus research efforts, reiterating concerns about sustainable R&D funding. Details of the discussion are described in the conference proceedings (*7*).

### Considerations for the Next Henipavirus Conference

References to the intergovernmental negotiating body drafting and negotiating the pandemic agreement were absent from discussions at Hendra@30 (*27*). WHO member states recently concluded negotiations and voted to adopt the instrument during the World Health Assembly on May 19, 2025 (*28*). The agreement provides for targeted research over the next 5 years, benefiting henipavirus R&D and achievement of the WHO CORC paramyxovirus priorities. Articles 19 and 20 of the pandemic agreement address international cooperation and sustainable financing for pandemic prevention, preparedness and response (*29*); therefore, those are areas of opportunity for henipavirus researchers and international policymakers to establish dialogue and learn from each other.

Another area for improvement is ensuring timely access to medical countermeasures when needed by the affected populations. Because of time limitations, neither the Nipah@20 nor the Hendra@30 conference provided a specific forum to discuss access to medical countermeasures; nonetheless, the 2024 update to the Nipah disease R&D blueprint addressed several issues related to ensuring access (*5*). We propose that access be integrated into the discussions of the new *Paramyxoviridae* CORC, as well as in the planning of the next major henipavirus conference, possibly Nipah-Malaysia@30 in Malaysia (30 years after the first outbreak in 1998–1999) or Nipah-Bangladesh@25 in Bangladesh (25 years after the first outbreak in Meherpur, Bangladesh, in 2001).

Although Hendra@30 discussed ecologic countermeasures not contemplated in its Nipah@20 predecessor, it missed the opportunity to host representatives from national regulatory agencies in henipavirus-affected countries, who were invited but were unable to travel; having those experts will be considered in planning the next conference. To ensure participation from stakeholders involved in reviewing data and regulating licensure of, and ultimately access to, medical countermeasures for the persons who need them most, one possibility will be to conduct hybrid science and policy interface sessions, similar to sessions conducted during the 8th World OneHealth Congress (*30*).

In terms of basic science, 2 presentations addressed the topic of cell-mediated immunity (CMI), and a few more addressed innate immunity indirectly. Encouraging more presentations on immunity will be an area to consider for the next meeting; we anticipate a full session dedicated to CMI, including vaccine-elicited immunity and CMI-elicited immunity by natural infection among NiV and HeV infection survivors in Bangladesh, Malaysia, India, and Australia. One intention is to ensure greater representation from the Philippines; the henipavirus R&D community has much to learn from epidemiologic studies and from survivors of the Nipah-like outbreak in that country.

We anticipate that ex vivo models, including organoids and organs-on-a-chip, will become a booming area of research in the coming years, because that topic was of great interest during Hendra@30. As noted in the proceedings, 1 poster and 4 oral presentations at the conference featured the use of reconstituted airway epithelia as a model for the study of viral replication and pathogenesis; the development of human cerebral organoids, or 3-dimensional, self-organizing tissue-like structures derived from human induced pluripotent stem cells; organ-on-a-chip micro physiologic models to emulate the alveoli and capillaries of the human lung; the use of a 3-dimensional cortical organoid model of human cerebral cortex; and the use of primary normal human bronchial epithelial cells grown and cultured in transwells at air–liquid interfaces.

Last, we anticipate that major topics at the next meeting will be, again, bat ecology, human behavior, and the design of strategies to prevent spillover. Furthermore, given that many novel henipa-like viruses are increasingly being detected in shrews, a specific focus on evolutionary ecology of henipaviruses in bats versus other species could be informative. We recommend that ecologic countermeasures be at the forefront of henipavirus prevention. We will continue to advocate for the design and implementation of these ecologic countermeasures and the fundamental ecology studies that inform them as part of a One Health strategy (*29*). That approach will ensure that primary and secondary prevention remains in the equation of pandemic prevention, preparedness and response, as enshrined in the WHO Pandemic Agreement.
